# CREB3L1 promotes tumor growth and metastasis of anaplastic thyroid carcinoma by remodeling the tumor microenvironment

**DOI:** 10.1186/s12943-022-01658-x

**Published:** 2022-10-03

**Authors:** Zongfu Pan, Tong Xu, Lisha Bao, Xiaoping Hu, Tiefeng Jin, Jinming Chen, Jianqiang Chen, Yangyang Qian, Xixuan Lu, Lu li, Guowan Zheng, Yiwen Zhang, Xiaozhou Zou, Feifeng Song, Chuanming Zheng, Liehao Jiang, Jiafeng Wang, Zhuo Tan, Ping Huang, Minghua Ge

**Affiliations:** 1 Center for Clinical Pharmacy, Cancer Center, Department of Pharmacy, Zhejiang Provincial People’s Hospital (Affiliated People’s Hospital, Hangzhou Medical College), Hangzhou, China; 2 Key Laboratory of Endocrine Gland Diseases of Zhejiang Province, Zhejiang Provincial People’s Hospital, Hangzhou, China; 3 Otolaryngology & Head and Neck Center, Cancer Center, Department of Head and Neck Surgery, Zhejiang Provincial People’s Hospital (Affiliated People’s Hospital, Hangzhou Medical College), Hangzhou, China; 4 Department of Clinical Pharmacy, College of Medicine, The First Affiliated Hospital, Zhejiang University, Hangzhou, China

**Keywords:** Anaplastic thyroid carcinoma, CREB3L1, Collagen, Extracellular matrix, Cancer**-**associated fibroblasts

## Abstract

Anaplastic thyroid carcinoma (ATC) is an extremely malignant type of endocrine cancer frequently accompanied by extrathyroidal extension or metastasis through mechanisms that remain elusive. We screened for the CREB3 transcription**-**factor family in a large cohort, consisting of four microarray datasets. This revealed that CREB3L1 was specifically up regulated in ATC tissues and negatively associated with overall survival of patients with thyroid cancer. Consistently, high expression of CREB3L1 was negatively correlated with progression**-**free survival in an independent cohort. CREB3L1 knockdown dramatically attenuated invasion of ATC cells, whereas overexpression of CREB3L1 facilitated the invasion of papillary thyroid carcinoma (PTC) cells. Loss of CREB3L1 inhibited metastasis and tumor growth of ATC xenografts in zebrafish and nude mouse model. Single**-**cell RNA-sequencing analysis revealed that CREB3L1 expression gradually increased during the neoplastic progression of a thyroid follicular epithelial cell to an ATC cell, accompanied by the activation of the extracellular matrix (ECM) signaling. CREB3L1 knockdown significantly decreased the expression of collagen subtypes in ATC cells and the fibrillar collagen in xenografts. Due to the loss of CREB3L1, ATC cells were unable to activate alpha**-**smooth muscle actin (α**-**SMA)**-**positive cancer**-**associated fibroblasts (CAFs). After CREB3L1 knockdown, the presence of CAFs inhibited the growth of ATC spheroids and the metastasis of ATC cells. Further cytokine array screening showed that ATC cells activated α**-**SMA**-**positive CAFs through CREB3L1**-**mediated IL**-**1α production. Moreover, KPNA2 mediated the nuclear translocation of CREB3L1, thus allowing it to activate downstream ECM signaling. These results demonstrate that CREB3L1 maintains the CAF**-**like property of ATC cells by activating the ECM signaling, which remodels the tumor stromal microenvironment and drives the malignancy of ATC.

**Graphical Abstract:**

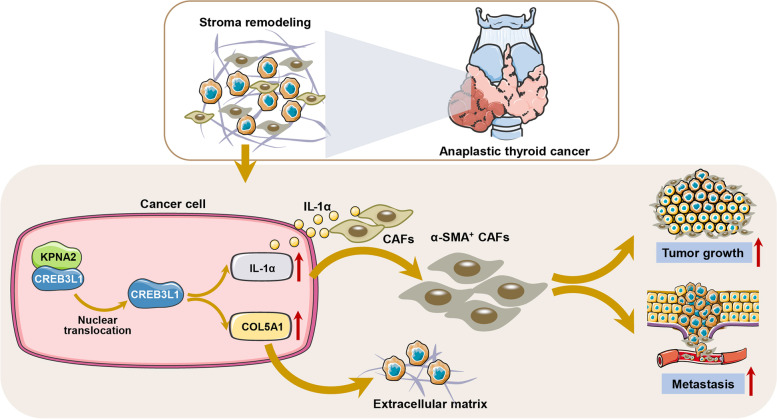

**Supplementary Information:**

The online version contains supplementary material available at 10.1186/s12943-022-01658-x.

## Background

Thyroid carcinoma is the most common malignant endocrine tumor, with its incidence gradually increasing in recent years [1]. Anaplastic thyroid carcinoma (ATC) is a rare but extremely malignant thyroid**-**cancer subtype. Patients with ATC are generally classified as being in stage IV, according to the tumor**-**node**-**metastasis (TNM) classification system of the American Joint Committee on Cancer (AJCC), and the median survival time is only 3–7 months [2]. Currently, there are no effective therapies that prolong patients’ overall survival [3–5]. ATC has an extremely rapid growth**-**rate and is highly invasive. More than 40% of ATC patients have distant metastases, and often die from asphyxia caused by local tumor expansion or distant metastasis [6].

The extracellular matrix (ECM) is a fundamental part of the tumor microenvironment. Biological and mechanical changes in the ECM profoundly affect tumor invasion, metastasis, immune escape, and drug resistance [7]. In a primary tumor mass, the ECM is strictly modulated in a tumor**-**permissive way, which in turn facilitates tumor progression and influences cancer cell invasion. By interacting with stromal cells, the tumor cells remodel the ECM, promoting covalent intermolecular cross**-**linkages and massive deposition of supramolecular aggregates such as fibrillar collagens [8–10]. Several collagen subtypes are closely associated with thyroid cancer occurrence and development. Type V collagen plays important roles in the adhesion, migration, and invasion of thyroid cancer cells [11]. Type I collagen is up**-**regulated in thyroid cancer and associated with the poor survival of its patients [12, 13]. A known characteristic of ATC is its ability to easily shape the tumor matrix microenvironment. Compared to that in papillary thyroid cancer (PTC) tissues, biological processes such as collagen synthesis and extracellular matrix remodeling are overstimulated in ATC tissues [14]. Given that ECM has a crucial regulatory effect on the malignant phenotypes of ATC, the mechanism that underlies ECM remodeling in ATC urgently needs to be elucidated.

The cAMP responsive element binding protein 3 (CREB3) family members locate in the endoplasmic reticulum (ER) membrane and act as transcription factors when cleaved by S1P and S2P proteases. In mammals, the CREB3 family consists of five members, which are essential for protein secretion, lipid metabolism, and survival. CREB3L1 acts as the regulatory protein of the thyroid stimulating hormone, thus, it controls the induction of collagen secretion in thyroid tissues [15]. Aberrant expression of CREB3L1 is thought to be the key driver of malignant progression of epithelioid fibrosarcoma, breast and bladder cancer [16–18]. In our previous study, we concluded that CREB3L1 was the master regulator of most collagen subtype genes in ATC, indicating that it likely regulates ECM signaling. However, the exact role and mechanism of CREB3L1 in ATC progression remain elusive.

Here, we showed that CREB3L1 is required for tumorigenesis and metastasis of ATC both in vitro and in vivo. It is translocated into the nucleus by KPNA2, where it activates the ECM signaling, thereby shaping the ATC tumor stromal microenvironment. These results confirm that CREB3L1 remodels the tumor stromal microenvironment, allowing us to newly identify CREB3L1 as the key driver of ATC progression.

## Methods

### Microarray information

The GSE29265, GSE33630, GSE65144, and GSE76039 datasets were acquired from Gene Expression Omnibus (GEO, https://www.ncbi.nlm.nih.gov/geo/). The single**-**cell RNA**-**sequencing (scRNA**-**seq) datasets, GSE148673 [19] and GSE134355 [20], containing ATC samples or healthy thyroid samples, were also acquired from the GEO database. The GSE29265 dataset contained 9 ATC, 20 PTC, and 20 normal tissue (NT) samples. The GSE33630 dataset contained 11 ATC, 49 PTC, and 45 NT samples [21]. The GSE65144 dataset contained 12 ATC and 13 NT samples [22] and the GSE76039 dataset contained 20 ATC and 17 poorly differentiated thyroid carcinomas (PDTC) samples [23, 24]. Seurat package was applied to merge and conduct a quality control of the scRNA**-**seq sample data in the R4.0.4 software. The Remove Batch Effect function of the limma package was used to correct the batch effect between the four datasets in the R4.0.4 environment. Our integrated dataset contained 216 thyroid tissue samples. The above**-**mentioned datasets were performed on the Affymetrix HT HG**-**U133^+^ PM Array platform.

### Survival analysis

The stage**-**plot analysis of CREB3L1 expression in normal thyroid tissues and thyroid tumors was obtained from the Gene Expression Profiling Interactive Analysis (GEPIA) database (http://gepia.cancer**-**pku.cn/) [25]. For survival analysis, the expression of CREB3L1 and COL5A1 in thyroid cancer was acquired from the Kaplan–Meier plot database (https://kmplot.com/analysis/). Using the survminer, survival, and My.setpwise packages in the R4.0.3 software, we conducted a univariate and multivariate Cox regression analysis of CREB3L1 and other potential risk factors. The thyroid cancer cohort was obtained from The Cancer Genome Atlas database.

### Gene Set Enrichment Analysis

The clusterProfiler package (3.18.0) for biological**-**term classification and enrichment analysis was used to perform Gene Set Enrichment Analysis (GSEA) to examine the CREB3L1**-**associated ECM and collagen signaling in ATC, in the R4.0.4 software environment. Based on the expression of CREB3L1, the GSEA generated the Kyoto Encyclopedia of Genes and Genomes (KEGG) enrichment results.

### Single cell copy number variation analysis and cell type annotation

The copy number variation analysis was performed on the scRNA**-**seq samples with the CopyKAT package in the R4.0.4 software, and used to annotate the diploid and aneuploid cells. The total gene expression of each cell was normalized to 10,000, and logarithmic normalization and linear regression scaling were performed. The FindVariableFeatures function identified the most variably expressed genes. We performed dimension reduction analysis on the principal components and corrected the batch effect with the principal components analysis and RunHarmony function, respectively. Both the FindNeighbors function and FindClusters functions were used for the clustering analysis. The UMAP method visually depicts the dimensionality reduction and clustering results. Finally, the SingleR and Celldex packages were used to annotate cell types.

### Analysis of cell trajectory and cell–cell interaction

A pseudo**-**temporal analysis, utilizing the reduceDimension function of the Monocle package and DDRTree method, conducted the cell trajectory. Then, the FindMarkers function explored the genes characteristic to all the different time series branches. In Python 3.8.5, the Cellphonedb software analyzed and visualized the interactions between the ATC**-**cell subgroups and other cells in the immediate microenvironment.

### ECM and collagen score

The lists of ECM and collagen genes were obtained from the Reactome database (Version 79, https://reactome.org/) and HUGO gene nomenclature committee database (https://www.genenames.org/), respectively (supplementary file S1). The enrichment scores of the above**-**mentioned genes in single cell sequences were calculated with the gene set variation analysis (GSVA) package z**-**score algorithm and visualized with the ggplot2 package in R4.0.4.

### Cell culture, transfection, migration, and invasion assays

The cell lines 8505C (DSMZ, Cat# ACC**-**219), BCPAP (DSMZ Cat# ACC**-**273), and Nthy**-**ori 3–1 (ECACC, Cat# 90,011,609) were cultured in an RPMI**-**1640 + 10% FBS (Gibco, Waltham, MA, USA) medium. The CAL62 cell line was obtained from DSMZ cell bank and cultured in a DMEM + 10% FBS (Gibco) medium. The immortalized thyroid cancer**-**associated fibroblast (CAF) cell line (China Center for Type Culture Collection, C2022119) was isolated from a human PTC tumor and cultured in a DMEM/F12 (1:1) (Gibco) medium, supplemented with 10% FBS.

The sgRNA CRISPR**-**Cas9 plasmids, which target CREB3L1, were obtained from Applied Biological Materials, and Lipofectamine 3000 (Thermo Fisher Scientific, Waltham, MA, USA) was used for transfection according to the manufacturer’s instructions. The plasmid pool contained three sgRNA sequences: TGGAACCCTTCCCGGCCGAC (sequence 1), GGACCACTTTACGGAGAACA (sequence 2), CGCCTGGATGCCTGGCGTAG (sequence 3). For small interference RNA (siRNA) transfection assay, cells were seeded in the 6**-**well plate with 30% confluence, and siRNAs were transfected with jetPRIME (Polyplus, Berkeley, CA, USA) at the final concentrations of 90 nM. The following siRNA sequences targeting KNPA2 were employed: sense sequence: 5’**-**CCUGGACACUUUCUAAUCU**-**3’ and antisense sequence: 5’**-**AGAUUAGAAAGUGUCCAGG**-**3’.

To evaluate the migration ability, a 250**-**μL pipette tip was used to scratch the confluent monolayers of the transfected cells. Cells were photographed at 0 h and 48 h post**-**scratching. To conduct the invasion assays, the transfected cells (5 × 10^4^ cells/well) were plated on 8**-**μm transwell inserts pre**-**coated with Matrigel (BD biosciences, Franklin Lake, NJ, USA), and cultured on a serum**-**free medium.

### qRT-PCR

Firstly, mRNA was extracted with the RNA**-**Quick Purification Kit (Esunbio, Shanghai, China). The mRNA was then reverse transcribed into cDNA by the Fast All**-**in**-**One RT Kit for qPCR (Esunbio). Next, 2 × Super SYBR Green qPCR Master Mix (Esunbio) was applied for PCR amplification and standardized to β**-**actin, the housekeeping gene. The primers used for the different genes are listed in Table 1.

**Table 1:** Primers for the different genes

Gene ID	Forward primer	Reverse primer
β-ACTIN	ACCTTCTACAATGAGCTGCG	CCTGGATAGCAACGTACATGG
CREB3L1	GGAGAATGCCAACAGGACC	GCACCAGAACAAAGCACAAG
COL1A1	CCCCTGGAAAGAATGGAGATG	TCCAAACCACTGAAACCTCTG
COL3A1	AAGTCAAGGAGAAAGTGGTCG	CTCGTTCTCCATTCTTACCAGG
COL4A1	TGTGGATCGGCTACTCTTTTG	TAGTAATTGCAGGTCCCACG
COL5A1	TCGCTTACAGAGTCACCAAAG	GTTGTAGATGGAGACCAGGAAG
COL6A1	AGGAGTCAAAGGAGCAAAGG	GCATTCACAGCAAGAGCAC
P4HA1	CCCCATTTTGACTTTGCACG	AACACTAGCTCCAACTTCAGG
INH1	ATCTTCCTGGTGCTGTTGAC	TGCTTGCGTGTTCCTATCAG
LEPRE1	TCACTGTCTTCAAAGCCCTC	GATGAGAGTAGGAAAAGTAGAGG

### Western blotting, nucleocytoplasmic separation, and co-immunoprecipitation

The ATC cells were lysed in a RIPA lysis buffer (Applygen, Beijing, China). The denatured protein samples were separated on a 10% SDS**-**PAGE gel and transferred to a PVDF membrane (Millipore, Burlington, MA, USA) which was blocked with 5% BSA in PBS with 0.1% tween for 60 min at 25℃, to reduce non**-**specific binding. Individual primary antibodies were introduced to the membrane and incubated overnight at 4℃. The primary antibodies included: CREB3L1 (sc**-**514635, Santa Cruz, Texas, USA), GAPDH (10,494–1**-**AP, Proteintech, Illinois, USA), COL5A1 (67,604–1**-**Ig, Proteintech), Histone**-**H3 (17,168–1**-**AP, Proteintech), Importin Beta (10,077–1**-**AP, Proteintech), KPNA2 (10,819–1**-**AP, Proteintech), and Lamin B1 (12,987–1**-**AP, Proteintech). The secondary antibodies were allowed to interact with the membrane for 60 min, at 26℃. The ECL (Fdbio, Hangzhou, China) was used for imaging. Nuclear**-**Cytosol Extraction Kit (P1200, Applygen) was used for the nucleocytoplasmic separation according to the instructions of the manufacturer.

To probe the interaction between the corresponding endogenous proteins, 8505C cells were lysed in an immunoprecipitation (IP) buffer (Beyotime, Shanghai, China) and, consequently, immunoprecipitated with the anti**-**CREB3L1 antibody (sc-514635, Santa Cruz). The Protein A/G Magnetic Beads (Selleck, Texas, USA) precipitated the target proteins, which were then washed three times with IP buffer, directly boiled in the loading buffer, and subsequently analyzed by gel electrophoresis and western blotting.

### Immunohistochemistry, immunofluorescence and Masson trichrome staining

Immunofluorescence staining and immunohistochemistry (IHC) protocols were carried out as previously reported [14], where 4**-**μm sections of human or xenograft paraffin**-**embedded tumor tissue were deparaffinized, rehydrated, and subjected to antigen retrieval in 1 mM EDTA (pH 8.0). Then, the endogenous peroxide was blocked with 0.3% hydrogen peroxide. To prevent non**-**specific binding, the sections were blocked with 5% goat serum in Tris**-**buffered saline to prevent non**-**specific binding. Primary antibodies were used in the following concentrations: anti**-**CREB3L1 antibody (1:200) and anti**-**COL5A1 (1:100). Biotinylated secondary antibodies were used for IHC while antibodies conjugated to Alexa Fluor® 488/594 were used for the immunofluorescence. The Masson Stain Kit (B1130, Applygen) was used for Masson trichrome staining according to the instructions of the manufacturer. The paraffin sections were first dewaxed and fixed, then stained with hematoxylin for 5 min and cleaned. Then, the slides were cleaned with hydrochloric acid ethanol, dehydrated with an alcohol series, 85%, 95%, 100% ethanol, for 3 min each, and sealed.

### Human inflammation array, flow cytometry, and ELISA

The treated ATC cells and CAFs (1 × 10^6^ cells each) were seeded in the upper (0.4 μm) and bottom compartment of tray, respectively, and incubated for 48 h. The supernatant was collected by centrifugation, and the human inflammation array was blocked with 1.5% BSA. The array was then incubated with a biotinylated detection antibody cocktail and streptavidin**-**conjugated fluor followed by incubation with the supernatants and standards. Finally, results were acquired by a gene microarray laser scanner and performing densitometry analysis according to the instructions (QAH**-**INF**-**3–1, RayBiotech, Atlanta, USA).

To generate the sphere, 1200 cells in a 30**-**μL drop were adsorbed on the lid of dishes and suspended culture for 3 days. Equal numbers of 8505C and CAFs were mixed when evaluating the cell–cell communication. To analyze the α**-**SMA**-**, FAP**-**, or PDGFRα**-** positive CAFs, found in the 8505C**-**derived sphere or co**-**culture with 8505C, the samples were digested and resuspended in PBS, containing 1.5% BSA and incubated with the primary antibodies anti**-**α**-**SMA (Cat#14,395–1**-**AP, Proteintech), anti**-**FAP (FAB3715A, R&D Systems, Minnesota, USA), and anti**-**PDGFRα (#567,950, BD biosciences), respectively. Then, samples were incubated with secondary antibody for 40 min at 25 °C. The cells and data were analyzed by the CytoFLEX (Beckman Coulter, Florida, USA) and FlowJo, respectively. To analyze the levels of cytokines IL**-**1α in the cell culture supernatants, human IL**-**1 alpha ELISA kit (RK00031, ABclonal, Wuhan, China) was performed according to the manufacturing instructions, specifically for detection.

### Animal models

All experiment on the animal tumor models were carried out according to the “Guide for the Care and Use of Laboratory Animals” (US National Institutes of Health, 8th edition), and the protocol was approved by the Animal Ethics Committee of the Zhejiang Provincial People’s Hospital. CREB3L1**-**knockdown 8505C cells (4 × 10^6^) were resuspended in 100 μL phosphate**-**buffered saline mixed with Matrigel® at a ratio of 10:1 (v/v), and subcutaneously injected into 6**-**week**-**old nude mice. We measured the body weight and tumor volume of these mice, once every 3–4 days. Tumor volume was calculated as tumor volume = Length × Width^2^/2. All mice were euthanized on day 35, and the tumors were harvested and photographed. For the nude mouse model of ATC pulmonary metastasis assay, treated cells (1 × 10^6^) were suspended in 100 µL PBS and injected into mice via the tail vein. Bioluminescence imaging was performed immediately after injection with 100 µl D**-**luciferin (3 mg/mouse). The bioluminescent imaging signals were used to monitor the lung metastases progression weekly, for 5 weeks.

Zebrafish were kept in a recirculating aquatic system at 28.5 °C, with a 12:12 h light/dark cycle, in accordance with standard practice, and fed dry pellets twice a day. For the tumor metastasis model, zebrafish embryos were mechanically dechorionated at 2 days post**-**fertilization (dpf), anesthetized with 0.15 mg/mL tricaine, and placed along plastic lanes immersed in 2.5% methylcellulose/PBS. The 8505C cells or CAFs were stained for 20 min at 37 °C, with DiI or DiO Cell**-**Labeling Solution (5 μg/mL, Beyotime). Unadulterated 8505C cells or 8505C cells combined with CAFs (1:1 ratio), all stained, were resuspended in PBS, loaded in a glass capillary needle, and microinjected into the perivitelline space (± 400 cells/embryo). Xenotransplanted embryos were then grown at 36 °C. The condition and progress of the embryos were monitored and documented daily, for three days. Thereafter, the embryos were pipetted for anesthesia, and then analyzed and photographed under a fluorescence microscope.

### Tissue samples

PTC samples were acquired from patients who underwent primary surgical resection between January 2006 and January 2010. At the time of assessment, pathological diagnosis and TNM classification were confirmed, according to the eighth AJCC recommendations. A total of 234 PTC specimens were collected and made into tissue microarrays to evaluate the prognostic correlation of CREB3L1. The clinicopathological information was listed in Table 2. Besides, non**-**tumorous tissues, PTC, and ATC were also collected to assess the CREB3L1 expression. All studies were approved by the Ethics Committee of Zhejiang Provincial People’s Hospital.

**Table 2:** Clinicopathological features of 234 patients with thyroid cancer

Variables	Stratification	CREB3L1 expression		*P*
		Low (*n* = 161)	High (*n* = 73)	
Age (years)	< 55	134	64	0.498
	≥ 55	27	9	
Gender	Men	39	13	0.356
	Women	122	60	
Bilaterality	Unilateral	131	54	0.265
	Bilateral	30	19	
Tumor number	Solitary	114	47	0.406
	Multiple	47	26	
Maximal tumor diameter	< 1 cm	36	16	0.847
	> 4 cm	12	4	
	1–4 cm	113	53	
Capsule invasion	Extracapsular	56	29	0.758
	Absent	87	36	
	Present	18	8	
Intrathyroidal dissemination	Absent	137	67	0.228
	Present	24	6	
T staging	pT1	83	35	0.89
	pT2	17	7	
	pT3	42	20	
	pT4	19	11	
N staging	pN0	59	29	0.718
	pN1a	54	26	
	pN1b	48	18	
M staging	M0	159	72	1
	M1	2	1	
Total thyroidectomy	Not done	127	49	0.077
	Done	34	24	
Lymph node dissection	Not done	9	6	0.636
	Done	152	67	

### Statistical analysis

All data of the three independent experiments are presented as the mean ± standard deviation (SD). The two**-**tailed Student’s t**-**test evaluated any significant differences between two groups, while differences between multiple groups were calculated with a one**-**way analysis of variance. The statistical significance was set as *P* < 0.05.

## Results

### CREB3L1 is highly expressed in aggressive thyroid cancer and correlates with poor prognosis

**Fig. 1:**
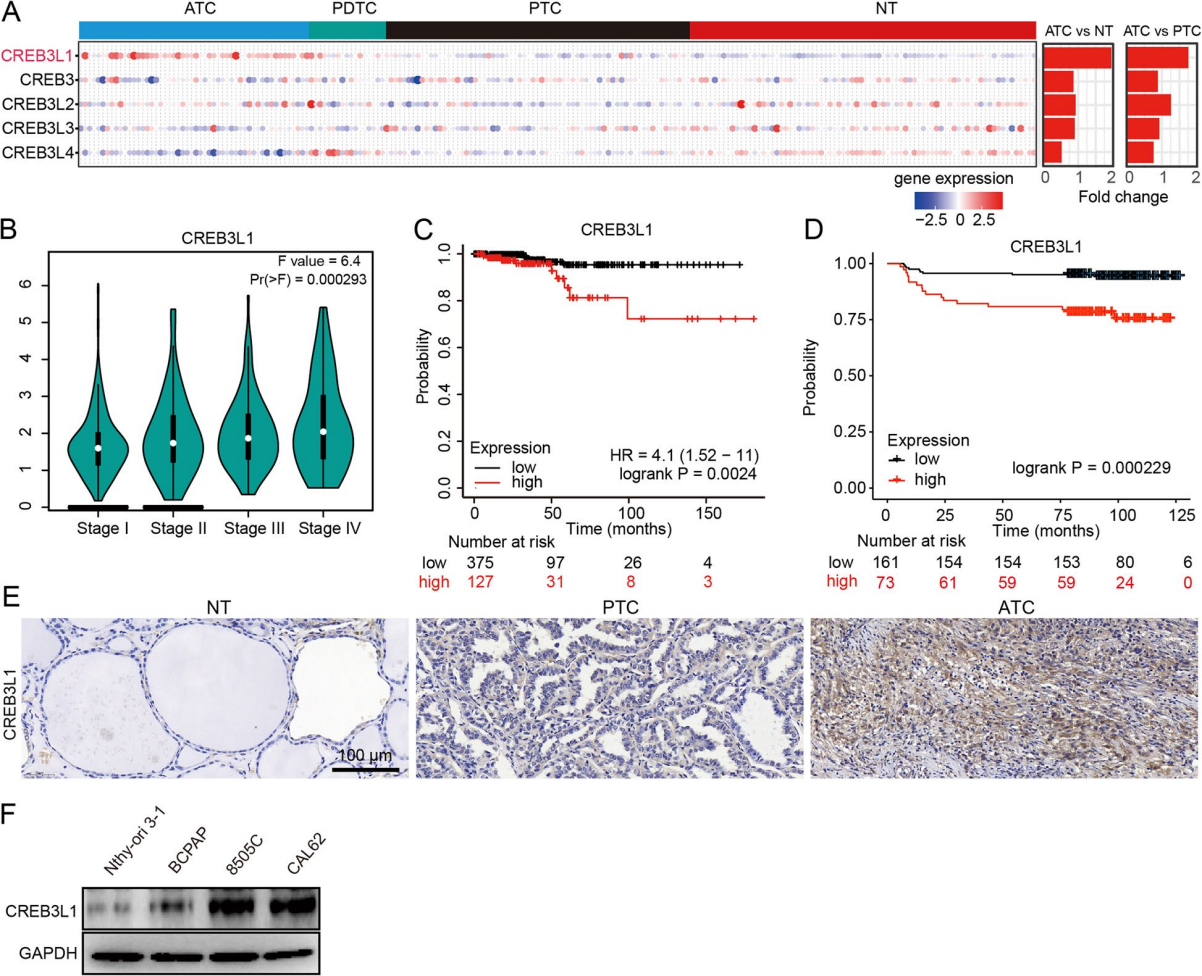
Expression of CREB3L1 in thyroid cancer and its clinical prevalence. **A** The expression of CREB3 family members in thyroid cancer subtypes and normal tissues was analyzed in four integrated datasets. **B** The relationship between CREB3L1 expression and tumor stage in thyroid cancer was retrieved from the GEPIA database. **C** Overall survival (OS) analysis of CREB3L1 in thyroid cancer was retrieved from the Kaplan–Meier plot database. **D** Progression**-**free survival analysis of CREB3L1 in an independent thyroid cancer cohort. Immunohistochemistry (IHC) staining of CREB3L1 was conducted in tissue microarrays contained 234 PTC samples. **E** IHC staining were used to analyze CREB3L1 expression in NT, PTC, and ATC tissues. **F** CREB3L1 expression in different thyroid cancer cell lines was analyzed by western blotting (WB). Data are presented as the mean ± standard deviation (SD). *P < 0.05, **P < 0.01 versus the respective NT, PTC, or ATC

To study the role of CREB3 family members in thyroid cancer, we integrated and analyzed 216 samples (78 NT, 69 PTC, 17 PDTC, 52 ATC) from four datasets (GSE33630, GSE65144, GSE29265, GSE76039). The results showed that only CREB3L1 was significantly up regulated in ATC tissues (Fig. 1A). CREB3L1 levels increased with tumor stage, and patients with high CREB3L1 expression were at a greater risk of death (Fig. 1B**-**C). According to the univariate analysis, thyroid cancer patients with elevated CREB3L1 expression (hazard ratio [HR] = 4.831, 95% confidence interval [CI]: 1.754–13.3, *P* = 0.00231) were more likely to die than those with lowered CREB3L1 expression (Table 3). Moreover, we also identified significant correlations between overall survival (OS) and T (*P* = 0.00217), M (*P* = 0.03159), and TNM staging (*P* = 0.0001331), maximal tumor diameter (*P* = 0.0137) and history of neoadjuvant treatment (*P* = 0.0001871). The multivariate Cox regression analysis indicated that both TNM staging and high CREB3L1 expression were accurate OS predictors in thyroid cancer patients (Table 3). IHC staining of CREB3L1 in an independent cohort of 234 thyroid cancer tissues showed that high expression of CREB3L1 was significantly correlated with shorter progression**-**free survival (*P* = 0.000229) (Fig. 1D). Furthermore, CREB3L1 was increased in ATC compared to PTC or NT in independent tissue samples (Fig. 1E). A similar trend was observed in thyroid cancer cell lines (Fig. 1F).

**Table 3:** Univariate and multivariate Cox regression analysis of CREB3L1 expression with patient prognosis

Variable	Univariate analysis		Multivariate analysis	
	HR (95% CI)	*P*-value	HR (95% CI)	*P*-value
Age	1.311e + 09 (0-inf)	0.9968		
Gender	1.896 (0.686–5.241)	0.2174		
T staging	2.629 (1.417–4.876)	0.00217^*^		
N staging	1.463 (0.4777–4.483)	0.5051		
M staging	5.443 (1.161–25.52)	0.03159^*^		
TNM staging	2.434 (1.542–3.842)	0.0001331^*^	3.621 (1.388–9.447)	0.00853^*^
Maximal tumor diameter	1.403 (1.072–1.836)	0.0137^*^		
Lymph node number	1.003 (0.9068–1.11)	0.9527		
Primary neoplasm focus type	0.2495 (0.05635–1.105)	0.06745		
Iodine radiotherapy	4.005e + 08 (0-inf)	0.9991		
Lymph node preoperative scan indicator	1.496 (0.4241–5.273)	0.5313		
History of neoadjuvant treatment	18.24 (3.976–83.66)	0.0001871^*^		
CREB3L1 expression	4.831 (1.754–13.3)	0.00231^*^	4.979 (1.074–23.08)	0.04025^*^

*HR* Hazard ratio

^*^ Statistical significance

### CREB3L1 was required for supporting cancer cell invasion in ECM

**Fig. 2:**
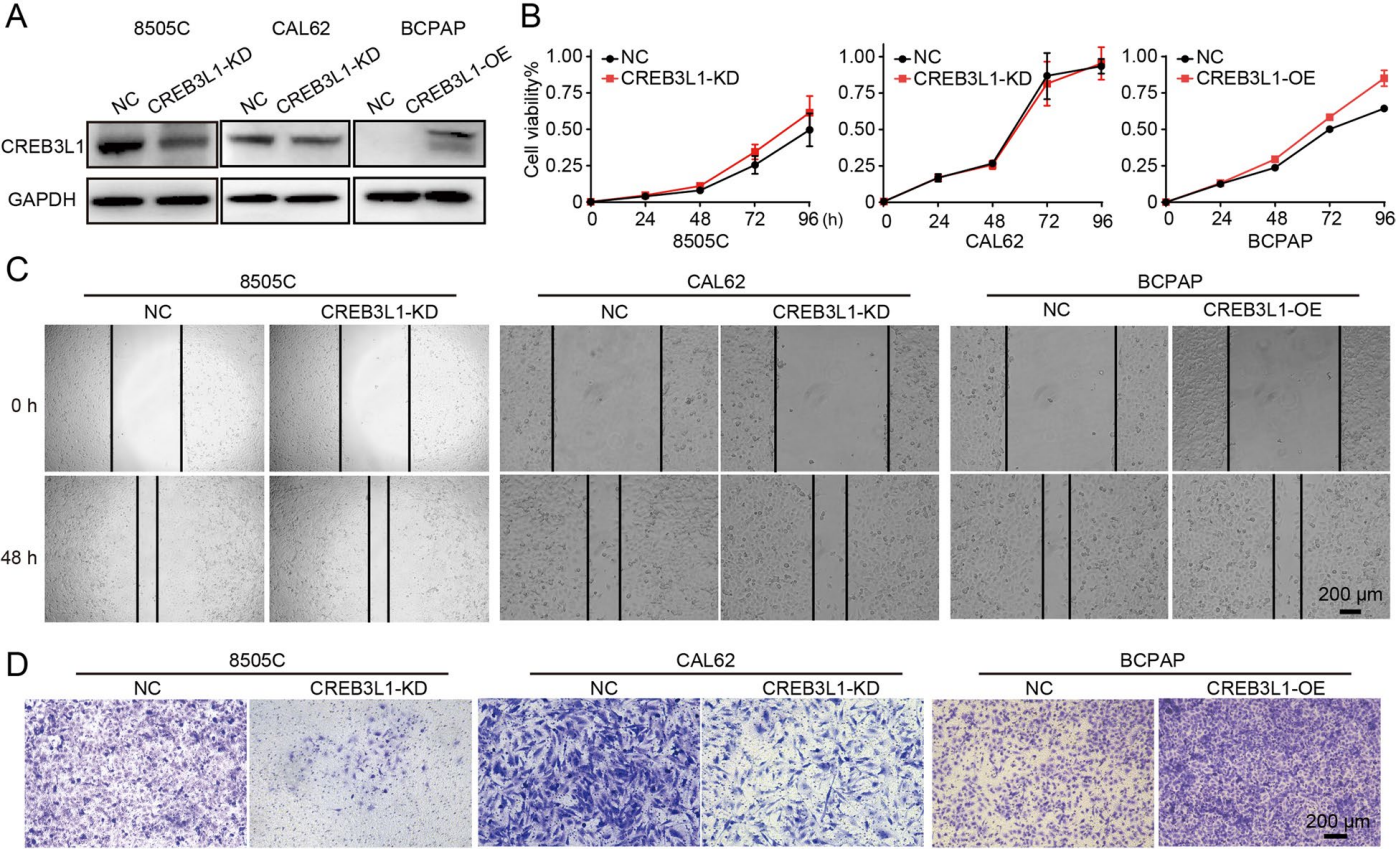
CREB3L1 knockdown suppressed the ATC invasion in vitro. **A** The knockdown or overexpression efficiency of CREB3L1 was analyzed by WB. Cell viability (**B**), migration (**C**), and invasion (**D**) were determined after CREB3L1 knockdown or overexpression in 8505C, CAL62, or BCPAP cells, respectively. Data are shown as the mean ± SD

Given that ATC was featured with rapid growth**-**rate and highly invasive behavior, we sought to determine whether high expression of CREB3L1 was responsible for maintaining the aggressive phenotype of ATC. CREB3L1 was knocked down or overexpressed with high efficacy, as confirmed by western blot (Fig. 2A). The deletion or overexpression of CREB3L1 did not obviously influence the proliferation and migration of ATC and PTC cells (Fig. 2B**-**C). However, CREB3L1 knockdown dramatically impeded ATC**-**cell invasion while CREB3L1 overexpression promoted PTC**-**cell invasion in the presence of Matrigel (Fig. 2D).

### Knockdown of CREB3L1 significantly inhibited the tumorigenesis and metastasis of ATC cells

**Fig. 3:**
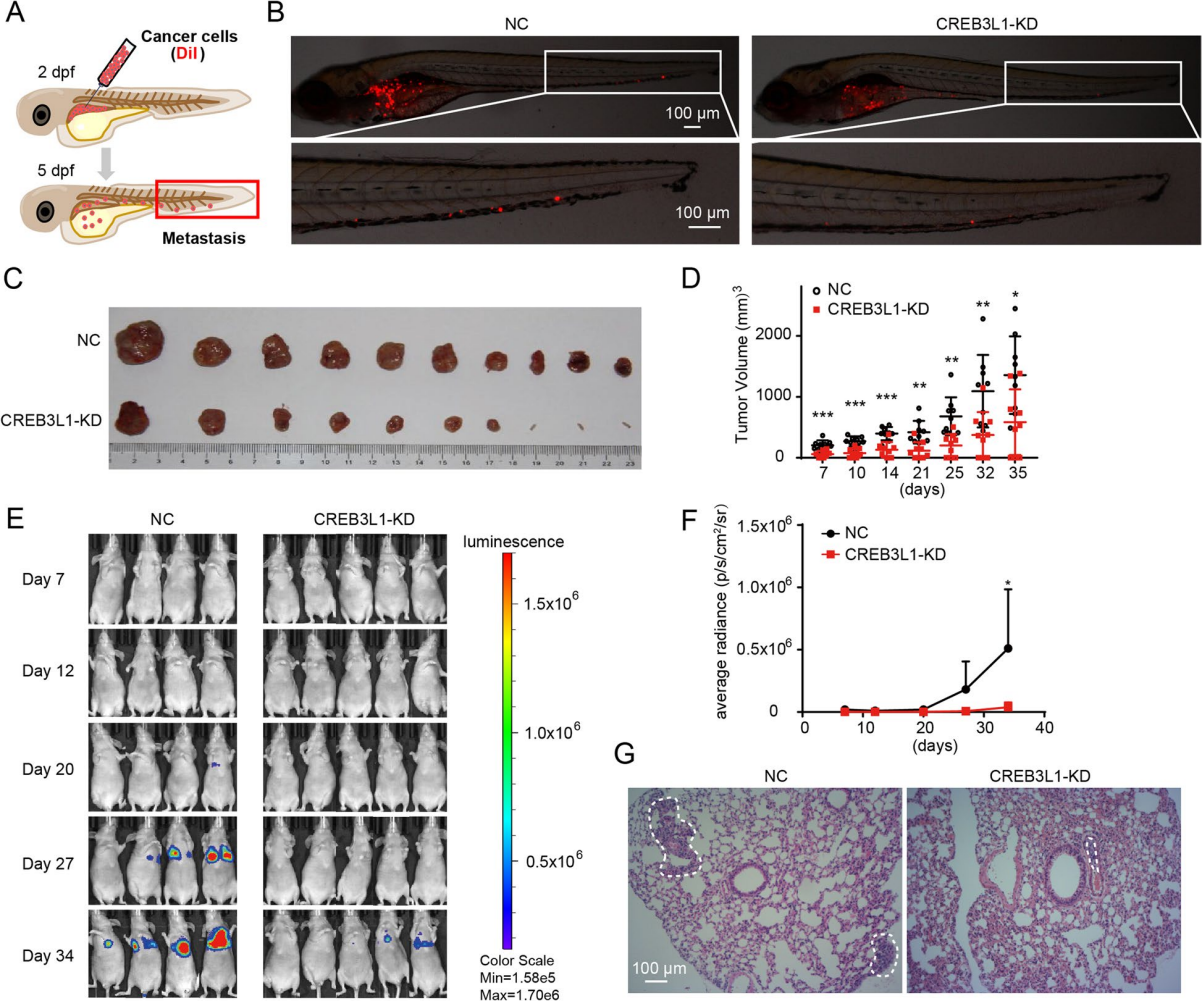
CREB3L1 knockdown decreased metastasis and tumor growth of ATC in vivo. **A** Schematic diagram of the experimental procedure. DiI**-**labeled 8505C cells (red) were implanted into the perivitelline space of each zebrafish. **B** The zebrafish xenograft model was employed to evaluate the metastatic ability of 8505C cells, after CREB3L1 knockdown. **C-D** CREB3L1 knockdown slowed tumor growth in nude mice with ATC xenografts. **E**–**F** CREB3L1 knockdown inhibited tumor metastasis in nude mice with intravenous injection of 8505C cells. **G** H&E staining revealed the ATC metastases (dotted**-**line circle) in the lung tissues. Data are shown as the mean ± SD. *P < 0.05, **P < 0.01, ***P < 0.001 NC versus CREB3L1**-**KD

To visualize the effect of CREB3L1 on ATC metastasis in vivo, a zebrafish xenograft model was employed. CREB3L1 knockdown obviously decreased the metastatic ability of the ATC cell**-**derived xenograft on 5 dpf (Fig. 3A**-**B). Further, to evaluate the effect of CREB3L1 expression on tumor growth in vivo, ATC cells with CREB3L1 knockdown were subcutaneously injected into nude mice (Fig. 3C**-**D). The mice bearing the CREB3L1**-**knockdown cell**-**derived xenografts, showed a substantially lower tumor volume, compared to that in the control mice. Moreover, an ATC pulmonary-metastasis mouse model was created by intravenous injection of 8505C cells. The metastatic intensity was dramatically reduced when CREB3L1 was knocked down in the 8505C cells (Fig. 3E**-**G). These data confirmed that CREB3L1 acts as a crucial regulator, which maintains the malignant phenotypes of ATC.

### High expression of CREB3L1 was associated with activation of ECM signaling

**Fig. 4:**
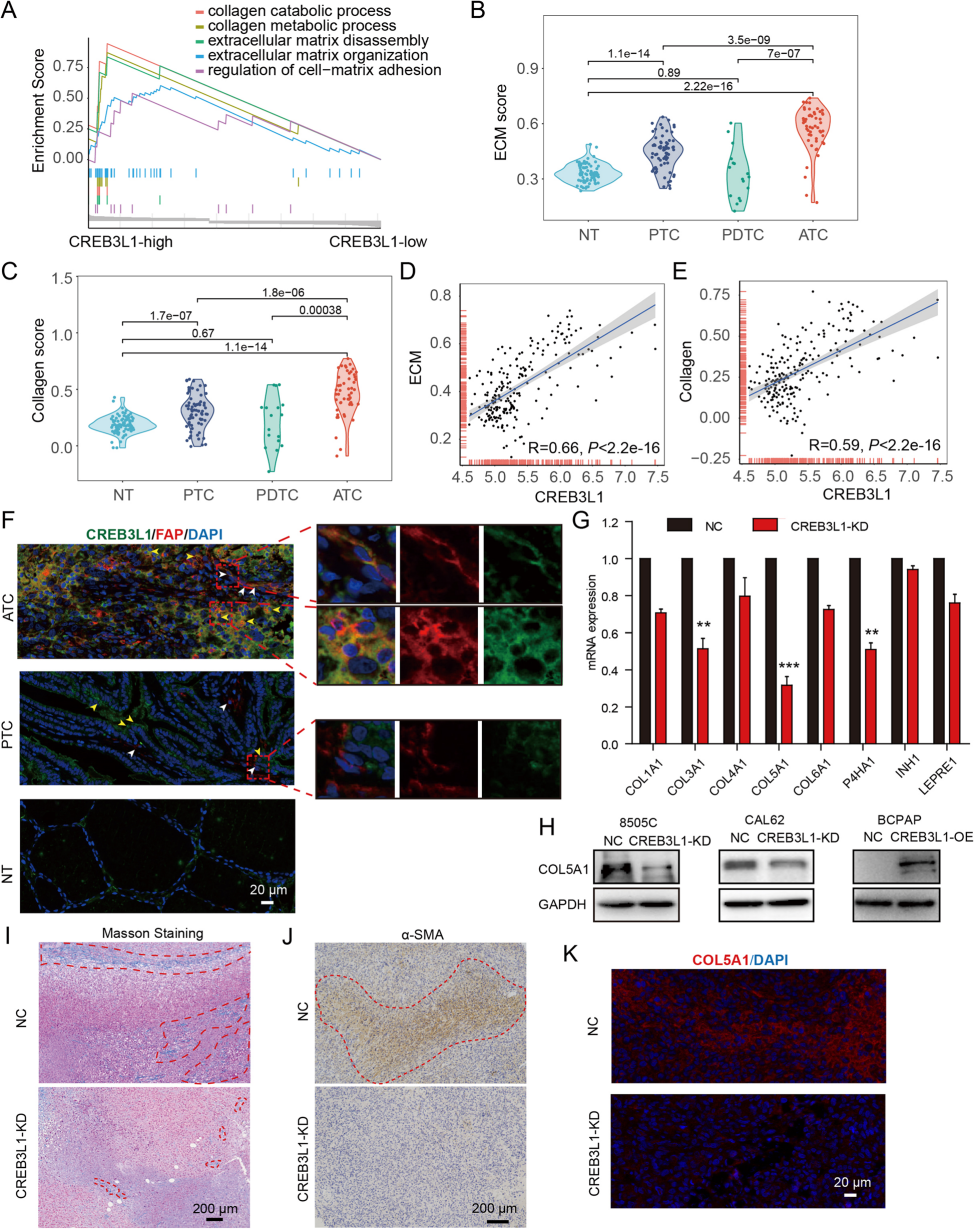
CREB3L1 is associated with ECM signaling in thyroid cancer. **A** GSEA determined the enrichment differences of biological processes depending on the CREB3L1 expression. **B-C** The evaluated activity scores of the extracellular matrix (ECM) and collagen signaling in NT, PTC and ATC samples. **D-E** The Pearson correlation analysis of CREB3L1 and ECM or collagen signaling. **F** Immunofluorescence staining of CREB3L1 and fibroblast marker FAP in thyroid cancer samples. **G** RT**-**PCR was used to detect the expression of collagen signals after CREB3L1 knockdown. **H** The expression of COL5A1 was detected, after CREB3L1 knockdown or overexpression in thyroid cancer cell lines. **I-J** After CREB3L1 knockdown, Masson trichrome staining and IHC staining were used to detect changes in collagen fibril abundance and the expression of α**-**SMA after CREB3L1 knockdown, respectively. **K** Immunofluorescence staining of COL5A1 in ATC cell-derived xenografts after CREB3L1 knockdown. Data are shown as the mean ± SD. *P < 0.05, **P < 0.01, ***P < 0.001 NC versus CREB3L1**-**KD

To delineate the mechanism of CREB3L1 in driving ATC aggressiveness, the GSEA analysis was conducted. The elevated expression of CREB3L1 significantly correlated with the activation of ECM and collagen signaling (Fig. 4A). A total of 216 samples from four datasets were further employed to determine the ECM and collagen score. Compared to NT or other thyroid cancer subtypes, ATC tissues had significantly activated ECM and collagen signaling (Fig. 4B**-**C). More importantly, the expression of CREB3L1 highly correlated with the activity of the ECM and collagen signaling (Fig. 4D**-**E).

Immunofluorescence staining showed that the expression of CREB3L1 was weak in normal and PTC tissues. Few fibroblasts infiltrated in the PTC tissues, and CREB3L1 was not expressed in FAP**-**positive fibroblasts (Fig. 4F). In contrast, massive FAP^+^ CREB3L1^+^ CAFs infiltrated in the ATC tissues. Additionally, numerous ATC cancer cells expressed both CREB3L1 and the fibroblast marker FAP, indicating ATC cells possessed a CAF**-**like phenotype. Further study confirmed that knockdown of CREB3L1 in ATC cells significantly inhibited the expression of various collagen isoforms and synthases, of which COL5A1 was most significantly down**-**regulated (Fig. 4G**-**H). In addition, COL5A1 levels increased with the progression of thyroid cancer and was negatively correlated with the OS of thyroid cancer patients (Figure S1A**-**B). COL5A1 levels were notably elevated in ATC, compared to that in NT and PTC tissues (Figure S1C). Knockdown of COL5A1 also inhibited the invasive ability of ATC cells (Figure S1D). The AnimalTFDB3 database predicted the binding site of CREB3L1 to be located in the promoter region of COL5A1 (Figure S1E). The dual**-**luciferase assay confirmed that CREB3L1 directly activated the transcription of COL5A1, while the COL5A1 mutant was not activated (Figure S1F). Furthermore, Masson trichrome staining and immunohistochemical staining of α**-**SMA confirmed that the loss of CREB3L1 significantly reduced collagen fibers and myofibroblasts in ATC cell**-**derived xenografts (Fig. 4I**-**J). Consistently, the abundance of COL5A1 in ATC cell**-**derived xenografts was also obviously declined after CREB3L1 knockdown (Fig. 4K).

### Aberrant expression of CREB3L1 and ECM signaling participated in the ATC evolving

**Fig. 5:**
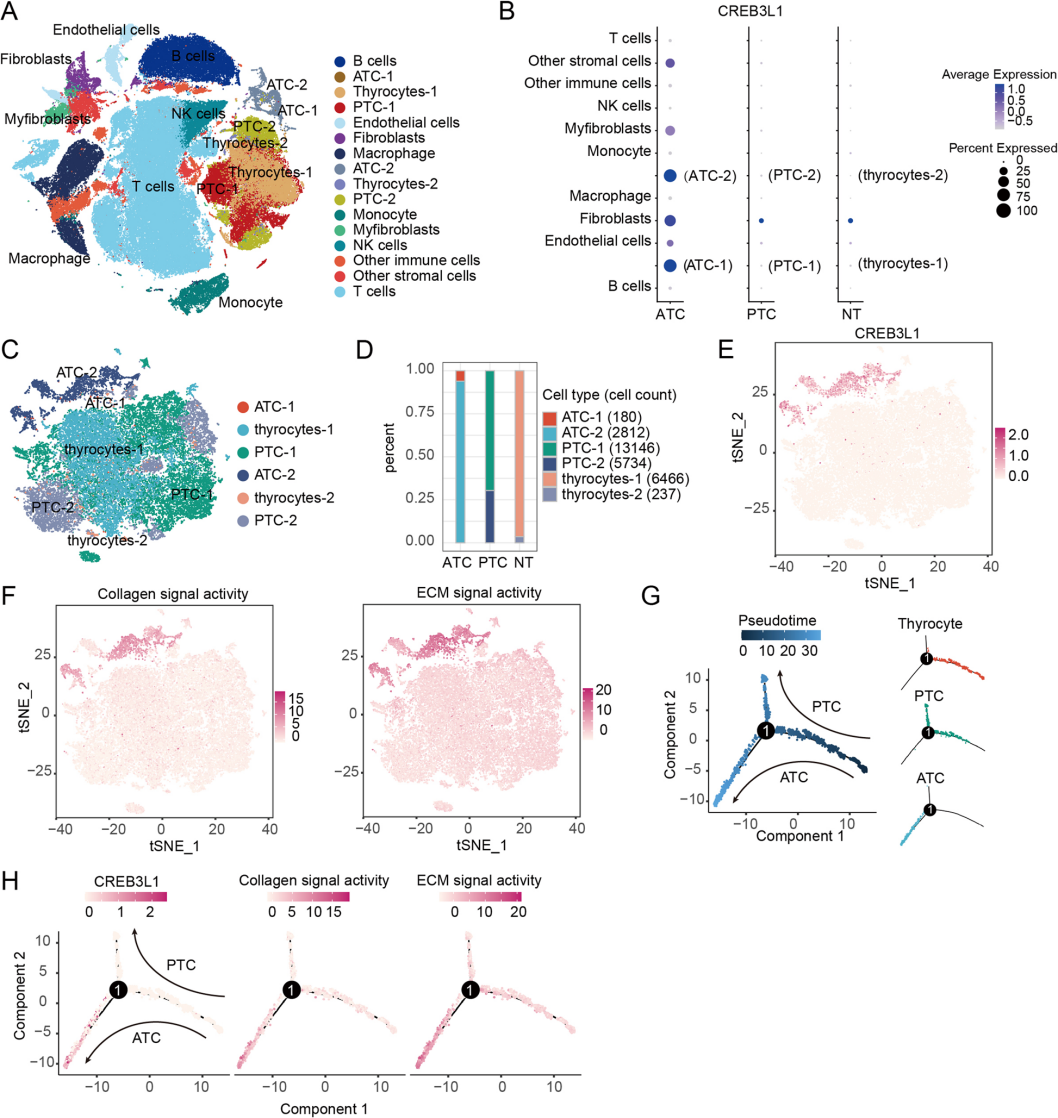
Single**-**cell RNA-sequencing revealed the role of CREB3L1 and downstream ECM signaling in ATC progression. **A** The UMAP plot of 16 cell subsets. **B** Expression, abundance, and positive ratio of CREB3L1 in different cell subsets. **C** Clustering results of thyroid follicular and cancer cells. **D** Proportion of different cell subsets in each group. **E** Expression and distribution of CREB3L1 in thyroid follicular cells and cancer cells. **F** Gene set variation analysis (GSVA) was used to calculate the activity of the ECM and collagen signaling in each cell. **G** Trajectory analysis of thyroid follicular and cancer cells. (H) Expression trends of CREB3L1, and the ECM and collagen signaling along the trajectory of ATC formation

To explore the role of CREB3L1 in maintaining the malignant phenotypes of ATC, two single**-**cell RNA-sequencing datasets were combined and analyzed. After quality control, a total of 130,226 cells and 16 cell subsets were obtained (Fig. 5A). CREB3L1 was highly expressed in ATC**-**1 and ATC**-**2 subpopulations and ATC**-**derived fibroblasts, but weakly expressed in PTC**-**derived fibroblasts and adjacent tissue (Fig. 5B). Further analysis of epithelial cells showed that notable presence of CREB3L1 in ATC tumor cells, which had extremely high ECM and collagen signaling activities (Fig. 5C**-**F). The trajectory analysis showed distinct branches of PTC or ATC cells that differentiated from thyroid epithelial follicular cells. Notably, the expression of CREB3L1 was dramatically up**-**regulated during evolution of ATC, accompanied by a discernably increased activity of ECM and collagen signaling (Fig. 5G**-**H). These results suggest that CREB3L1**-**mediated ECM signaling is essential for ATC formation and progression.

### CREB3L1 is involved in inducing differentiation of α-SMA^+^ CAFs

**Fig. 6:**
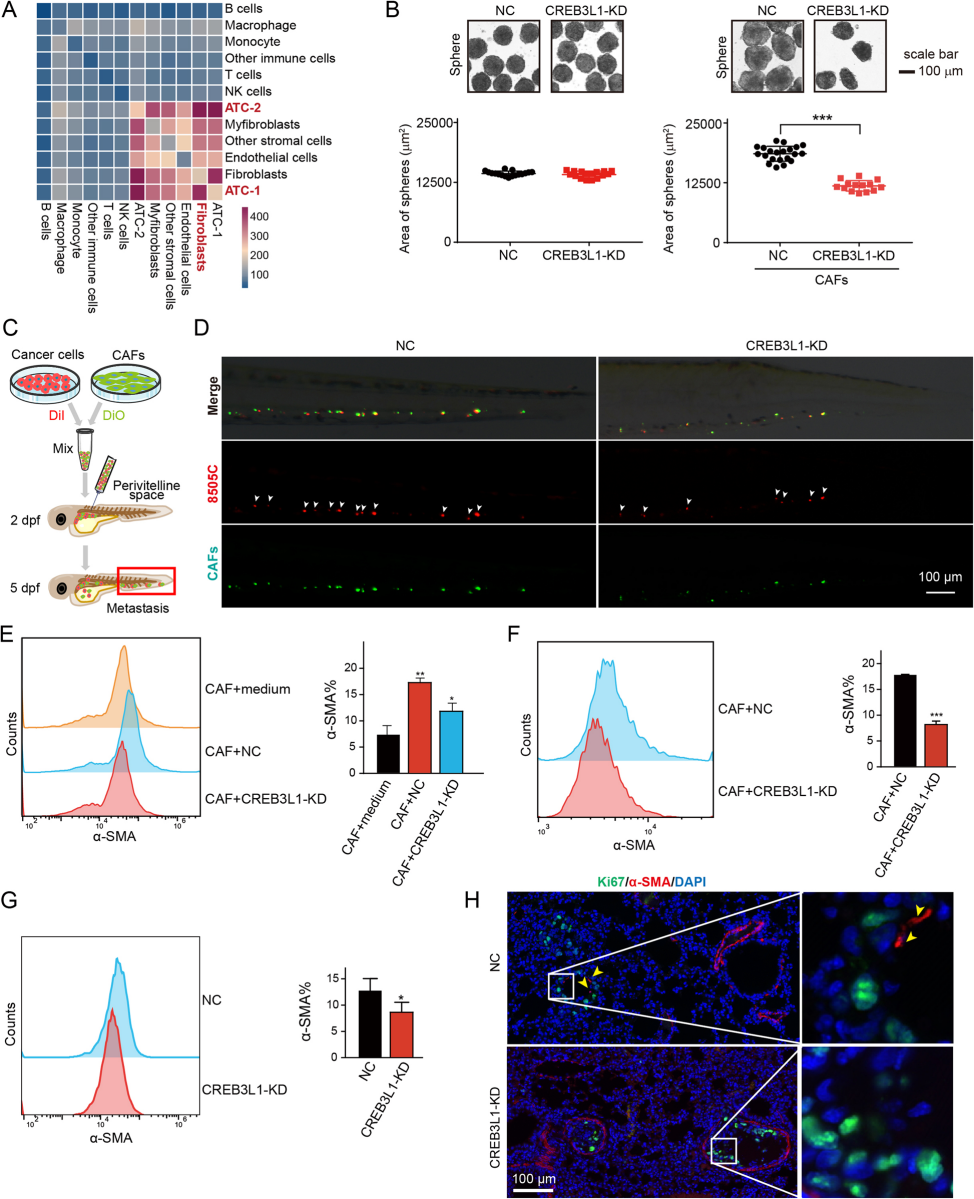
CREB3L1 is involved in remodeling the stromal microenvironment of ATC. **A** Single**-**cell RNA-sequencing was used to examine the interaction between ATC cell subsets and cells in the tumor microenvironment. **B** The sphere formation assay was used to detect the growth of 8505C cells mixed with or without CAFs after CREB3L1 knockdown. ***P < 0.001 CAF + NC versus CAF + CREB3L1**-**KD. **C** Schematic diagram of the experimental procedure. Equal numbers of DiI**-**labeled 8505C cells (red) and DiO**-**labeled CAFs (green) were mixed and implanted into the perivitelline space of each zebrafish. **D** The zebrafish xenograft model was employed to evaluate the CAF**-**mediated metastasis of 8505C cells after CREB3L1 knockdown. **E** Flow cytometry analysis of α**-**SMA**-**positive fibroblasts, after co**-**culture with 8505C cells. *P < 0.05, **P < 0.01 versus the respective CAF + medium, CAF + NC, or CAF + CREB3L1**-**KD. **F** Flow cytometry analysis of α**-**SMA positive fibroblasts in the sphere derived from the mixture of 8505C and CAFs. **G** Flow cytometry analysis of α**-**SMA**-**positive fibroblasts in the lung tissues of mice with ATC pulmonary metastasis. *P < 0.05 NC versus CREB3L1**-**KD. **H** Counterstaining of Ki67 and α**-**SMA with antibodies that specifically reacted with the human (Ki67) and mouse (α**-**SMA) antigen, respectively. Data are presented as the mean ± SD

Cancer cells stimulated the differentiation of stromal progenitors into CAFs, and their reciprocal interaction drove the ECM remodeling, creating a cancer niche that supported tumor growth and metastasis. Given that CREB3L1 was closely associated with ECM signaling, we investigated whether CREB3L1 was involved in shaping the tumor stromal microenvironment. The scRNA-seq analysis revealed that ATC**-**1 and ATC**-**2 subsets, fibroblasts, and myofibroblasts had frequent cell–cell interactions (Fig. 6A). Interestingly, knockdown of CREB3L1 in 8505C cells did not influence the sphere formation, while 8505C**-**derived spheres with CREB3L1 knockdown, in the presence of CAFs, were significantly smaller than that of the control group (Fig. 6B). This suggests that CREB3L1 was required for shaping CAFs to support the growth of ATC cells. Moreover, a mixture of 8505C cells and CAFs, implanted into the perivitelline space, facilitated the metastasis of ATC cells, while knockdown of CREB3L1 in 8505C cells dramatically reduced the metastatic number of ATC cells (Fig. 6C**-**D). Flow cytometry analysis showed that an ATC cell co**-**culture generated α**-**SMA^+^ CAFs, which was significantly diminished after CREB3L1 knockdown in 8505C cells (Fig. 6E). A similar result was also found in the sphere derived from the mixture of 8505C and CAFs (Fig. 6F). Other fibroblast markers such as FAP**-** and PDGFRα**-** positive CAFs did not obviously change after loss of CREB3L1 in 8505C cells (Figure S2), indicating α**-**SMA^+^ CAFs is a major subpopulation that regulated by CREB3L1. Additionally, we noted the considerably reduced proportion of α**-**SMA^+^ CAFs in the lung tissue of the CREB3L1**-**knockdown ATC pulmonary metastasis mouse group (Fig. 6G). Most ATC metastases colonized at a distance way from vasculatures and were frequently co**-**occurrent with α**-**SMA^+^ fibroblasts (Fig. 6H). In contrast, CREB3L1 knockdown attenuated the invasive ability of ATC cells, most of which, along with a few α**-**SMA^+^ fibroblasts, localized to the vasculatures (Fig. 6H).

### CREB3L1 facilitates IL-1α production to stimulate α-SMA^+^*CAFs differentiation*

**Fig. 7:**
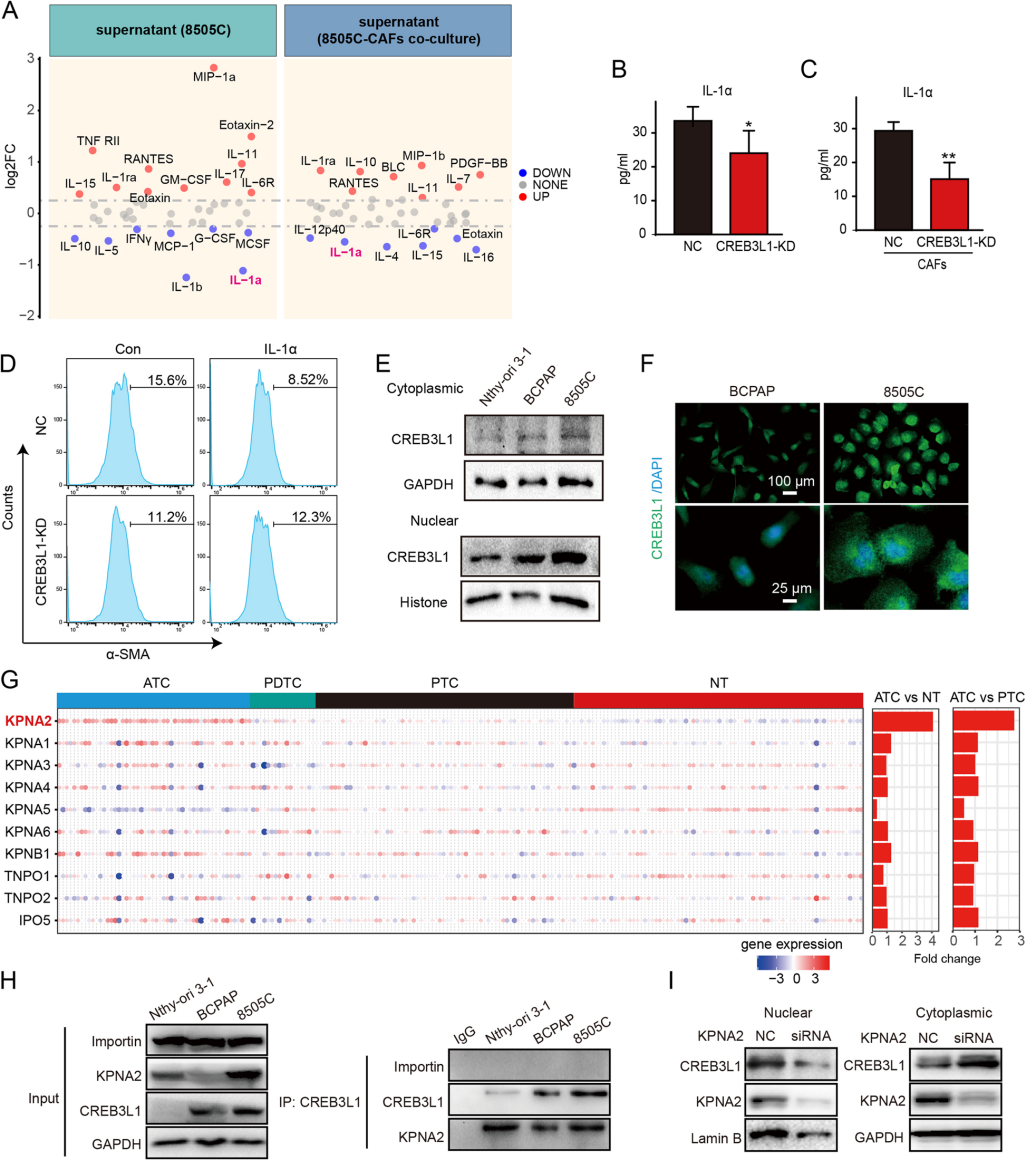
Nuclear translocation of CREB3L1 by KPNA2 activates IL-1α expression. **A** The cytokines changed in the supernatant of 8505C cells (left panel) and co-culture supernatant of 8505C-CAFs (right panel) after CREB3L1 knockdown were detected by human cytokine array. **B-C** Validation of IL-1α in the supernatant by ELISA kit. **D** The proportion of α-SMA^+^ CAFs was analyzed by flow cytometry. CAFs were co-cultured with 8505C cells and were exposed to 1 ng/mL IL-1α for 48 h. *P < 0.05, **P < 0.01 NC versus CREB3L1-KD or CAF + NC versus CAF + CREB3L1-KD. A nucleocytoplasmic separation assay (**E**) and immunofluorescence (**F**) were used to detect the localization of CREB3L1 in PTC and ATC cell lines. (**G**) The expressions of the nuclear transport receptor karyopherin family members were analyzed in four integrated datasets. **H** Co-immunoprecipitation was used to verify the interaction between CREB3L1 and KPNA2 in different thyroid cancer cells. **I** WB was used to detect the cytoplasmic and nuclear expression of CREB3L1 after KPNA2 silencing in 8505C cells

To explore the exact signal that induced the differentiation of α-SMA^+^ CAFs by ATC cells, a cytokine array was exploited to profile the change of cytokines in the supernatant derived from 8505C cells or from a co-culture with CAFs after CREB3L1 knockdown (Fig. 7A). Eight cytokines in the 8505C-derived supernatant and seven cytokines in the 8505C-CAFs co-culture supernatant were decreased at the threshold of 1.2-fold, after CREB3L1 knockdown. IL-1α was consistently downregulated in both experiments and was thus considered a candidate cytokine (Fig. 7A). The ELISA results confirmed that IL-1α was downregulated after CREB3L1 knockdown, when co-cultured with/without CAFs (Fig. 7B-C). Exogenous IL-1α stimulation did not promote the α-SMA^+^ CAF differentiation in the presence of 8505C cells. However, the simultaneous knockdown of CREB3L1 in 8505C cells and IL-1α stimulation reversed the α-SMA^+^ CAF reduction (Fig. 7D), suggesting an intricate signal for ATC cell-induced CAF activation.

Though CREB3L1 was regarded as a transcription factor after cleavage by S1P and S2P, the mechanism of how CREB3L1 mediated ECM activation remained elusive. The nucleocytoplasmic separation and immunofluorescence staining revealed the escalated nuclear translocation of CREB3L1 in ATC cells (Fig. 7E-F). When analyzing the amino acid sequence of CREB3L1, we noticed a nuclear localization sequence, located between amino acids 292 and 314 (Figure S1G). This suggested that transporter proteins regulate the nuclear entry of CREB3L1. Therefore, we screened the expression profiles of the karyopherin members of the nuclear translocation receptor superfamily. The results revealed that KPNA2 levels are significantly elevated in ATC tissues, compared to that in PTC and NT (Fig. 7G). Furthermore, co-IP results showed that KPNA2, but not importin, formed a complex with CREB3L1, and the binding level was the highest in ATC cells (Fig. 7H). We also found that KPNA2 knockdown significantly inhibited the nuclear translocation of CREB3L1, resulting in its retention of CREB3L1 in the cytoplasm (Fig. 7I). Collectively, these results suggest that nuclear translocation of CREB3L1 mediated by KPNA2 enhanced the ECM signal, thereby driving the malignant phenotype of ATC cells.

## Discussion

ATC is featured with aggressive local disease, high metastatic rate, and a rapidly fatal prognosis. ATC has a more vigorous epithelial-mesenchymal transition progress than PTC, which becomes the killing switch. The mesenchymal characteristics of ATC potentiates its gross extrathyroidal extension and metastasis. Previous studies have tried to uncover the mechanism that ameliorates ATC aggressiveness by comprehensively analyzing the master regulators and regulatory network [14, 26]. Several transcription factors including E2F7, FOXM1, NFYB, and CREB3L1 were significantly associated with the OS of thyroid cancer patients and may be the main regulators of ATC progression. However, the molecular mechanism that enhances the aggressive behavior of ATC, is still unclear. In the present study, the CREB family member CREB3L1 was identified as the key regulator that drives ATC aggressiveness. Based on the integrative analysis of multi-omics and multiple tumor models, we revealed that CREB3L1 facilitated the growth and metastasis of ATC tumors by activating the ECM signal and remodeling the tumor stromal microenvironment. The trajectory analysis showed that CREB3L1, accompanied by ECM signaling, is essential for the evolution of ATC. CREB3L1 expression in ATC cells facilitated IL-1α-mediated CAFs differentiation, thereby remodeling the cancer niche. The malignant phenotype maintained by CREB3L1, was associated with the KPNA2-mediated transportation of CREB3L1 into the nucleus, which activated the downstream ECM signal.

CREB3L1 is a member of the CREB3 transcription factor family involved in important biological processes such as metabolism, differentiation, and protein secretion. When screening the expression profile of the CREB3 family, we found that CREB3L1 was specifically upregulated in ATC tissues compared to that in PTC and NT tissues. Nevertheless, the role and mechanism of CREB3L1 in ATC aggressiveness remained unclear. Murakami et al. demonstrated that CREB3L1-deficient mice exhibited severe osteopenia due to the reduced collagen I, which was related to the transcriptional activation of COL1A1 by CREB3L1 [27, 28]. The altered expression of CREB3L1 has been reported in several tumor types, and its roles in tumorigenesis regulation has gradually been discovered. CREB3L1 acts as the downstream signal of PERK to regulate the expression of collagen I and FN1 and promote the invasion and metastasis of breast cancer [16]. Reportedly, elevated CREB3L1 expression can indicate a higher risk of PTC recurrence and potentially plays a role in thyroid cancer dedifferentiation [29]. Other studies concluded that *EWSR1-CREB3L1* gene fusion drives the occurrence and development of sclerosing epithelioid fibrosarcoma [17]. Our studies showed that loss of CREB3L1 in various models, including zebrafish and mouse xenografts, dramatically attenuated the invasiveness, metastasis, and tumor growth of ATC tumors. Interestingly, we noticed that altered CREB3L1 expression did not affect cancer cell proliferation or ATC cell-derived sphere size, but suppressed tumor growth in vivo. Crosstalk between tumor cells and the tumor microenvironment is vital for tumor growth. It was reported that CAFs promoted the growth of breast and lung cancer by providing a survival niche for cancer stem cells [30], and stimulated the malignant progression of pancreatic and liver cancer by secreting cytokines or exosomes [31, 32]. These findings supported our hypothesis that a possible role of CREB3L1 in mediating cell–cell communication or stromal remodeling to promote tumor growth in vivo. Furthermore, CREB3L1 knockdown, in the presence of CAFs, inhibited the sphere formation. We also found fewer collagen fibers and myofibroblasts in ATC cell-derived xenografts after CREB3L1 knockdown, implying that CREB3L1 drives the aggressiveness of ATC, mainly through regulating the tumor stromal microenvironment.

The ECM provides a physical scaffold for surrounding cells and regulates cell behavior, which is crucial to the tumor microenvironment. Collagens are the main proteins present in the ECM. Interaction of the ECM with cells induces biochemical and biophysical signals, which are essential in cancer development [33]. Collagens regulate cancer progression by regulating the metastasis, invasion, and survival of cancer cells [34–36]. Aberrant expression of the ECM and cytokines genes was correlates with PTC aggressiveness and the shorter OS period of patients [37]. In our previous studies, we concluded that the ECM processes are stronger in ATC than in PTC [26]. The adhesion of cancer cells to the ECM, mediated by the integrin receptor, is a vital step for the development of ATC metastasis [38]. In our studies, we associated the high expression of CREB3L1 with abnormal ECM activation in ATC. Further, scRNA-seq revealed that CREB3L1 was significantly upregulated along the ATC branch and was accompanied by enhanced ECM signaling, suggesting a close interactive relationship between CRBE3L1 and ECM, which are jointly involved in the ATC occurrence and development. Interestingly, we found that type V collagen, not type I collagen, was the chiefly suppressed collagen subtypes after CREB3L1 silencing, indicating that CREB3L1 directly activates the COL5A1 transcription. Moreover, COL5A1 negatively correlated with OS, and knockdown of COL5A1 inhibited the invasive ability of ATC cells.

Tumor cells frequently invoke phenotypic changes to stromal cells, thereby creating a cancerized niche, which supports tumor growth and metastasis. The reciprocal interplay between tumor cells and CAFs governs the architecture of the ECM [39]. Notably, we found that both cancer cells and CAFs had high expression of FAP and CREB3L1 in ATC tissues. ATC cancer cells showed a CAF-like property and the co-culture of ATC cells and CAFs significantly increase the proportion of α-SMA^+^ CAFs. The latter are activated fibroblasts that share similar properties with myofibroblasts [40, 41]. The cancer-associated myofibroblasts hijacked low metastatic cancer cells for metastasis in the presence of CXCL3-CXCR2 signaling [40]. Since α-SMA^+^ CAFs are involved in ECM remodeling and metastasis, this subpopulation is tightly associated with tumor progression and the poor prognosis of patients with cancer [42, 43]. Additionally, our present study revealed that IL-1α, a CAF activation cytokine, is dramatically elevated in ATC cells in our present study and was regulated by CREB3L1. Ishimoto et al*.* revealed that IL-1α, in addition to other inflammatory cytokines, induced the transformation of non-cancerous fibroblasts into CAFs by activating RHBDF2/TGFβ signaling and enhancing CAF motility, thereby stimulating diffuse-type gastric cancer cells to invade the ECM and lymphatic vessels [44]. A recent study also supported that metastasis-initiating cells secrete IL-1α and IL-1β, inducing lung fibroblast CXCL9 and CXCL10 production, fueling the colonization of lung metastases, ultimately creating a fibroblast niche [45]. In our present study, IL-1α stimulation did not promote the differentiation of α-SMA^+^ CAFs in the co-culture system. However, its stimulation, in addition to the simultaneous knockdown of CREB3L1 in ATC cells, overturned the decrease of α-SMA^+^ CAFs. Since the heterogeneity and plasticity of CAFs are notably prominent in cancers, CAFs are interchangeable depending on their location and exposure in the tumor [46]. More evidence is needed to delineate the intricate communications between ATC cells and CAFs.

The nuclear translocation of CREB3L1 enables it to activate ECM signaling directly through an intricate mechanism. Our results showed that the nuclear translocation is significantly higher in ATC cells than that in PTC or NT cells. Although CREB3L1 contains a nuclear localization sequence, little is known about how it is translocated into the nucleus. The nuclear transport receptor superfamily is a protein group involved in selective nuclear pore transport, in which KPNAs and KPNBs can transport proteins into the nucleus. The abnormal expression of nuclear transport receptors is involved in the malignant progression of tumors [47–49]. Our research elucidated that KPNA2 is specifically up-regulated in ATC tissues and strongly binds with CREB3L1 in the ATC cells. Loss of KPNA2 hindered the nuclear translocation of CREB3L1. Since the role of KPNA2 in thyroid cancer is rarely reported, our findings shed light on the KPNA2-mediated ECM remodeling in ATC by transporting CREB3L1 into the nucleus.

## Conclusions

Our findings recognize the crucial role of CREB3L1 in driving invasion, metastasis, and growth of ATC tumors by shaping the tumor stromal microenvironment. Nuclear transportation of CREB3L1, facilitated by KPNA2, directly activated the ECM signaling and resulted in the ECM remodeling of ATC. These insights provide important theoretical support for the occurrence and development mechanism of ATC and the ultimate identification of potential therapeutic targets.

## Supplementary Information

**Additional file 1: Figure S1.** CREB3L1 transcriptionally regulated the expression of COL5A1 to facilitate the aggressiveness of ATC. (A) The relationship of COL5A1 expression and tumor stages in thyroidcancer was analyzed. (B) Survival analysis of COL5A1 in thyroid cancer was analyzed. (C) IHC staining was used to analyze COL5A1 expression in NT, PTC and ATC tissues. (D) Transwell invasion assay was used to analyze the metastasis ability after the silence of COL5A1 in 8505C. (E-F) The AnimalTFDB3 database was used to predict the binding sequence of CREB3L1 to the COL5A1 promoter, and the Dual-luciferase assay to detect the transcriptional activation of CREB3L1 to COL5A1 wild-type or mutant binding sequence. (G) PredictProtein database was used to analyze the nuclear localization sequence of CREB3L1. Data shown are results of three independent experiments and shown as mean ± standard deviation(SD). *P < 0.05, **P < 0.01.

**Additional file 2: Figure S2.** Effect of CREB3L1 knockdown on the differentiation of CAFs. (A**-**B) Flow cytometry analysis of FAP or PDGFRα positive fibroblasts after co-culture with 8505C cells, respectively. CREB3L1 was knocked down in 8505C cells to evaluate its effect on the differentiation of CAFs.

**Additional file 3.** The list of ECM and collagen genes.

## Data Availability

The data supporting the conclusions of this paper have been provided in this paper and GEO database. In addition, all data for this study are available from the corresponding author upon reasonable request.

## Abbreviations

ATC: Anaplastic thyroid carcinoma
PTC: Papillary thyroid carcinoma
PDTC: Poorly differentiated thyroid carcinomas
CREB3L1: CAMP Responsive Element Binding Protein 3 Like 1
ECM: Extracellular matrix
CAFs: Cancer**-**associated fibroblasts
ER: Endoplasmic reticulum
KPNA2: Karyopherin Subunit Alpha 2
GEPIA: Gene expression profiling interactive analysis
GSEA: Gene set enrichment analysis
GSVA: Gene set variation analysis
KEGG: Kyoto encyclopedia of genes and genomes
scRNA**-**seq: Single**-**cell RNA-sequencing
FC: Fold change
GEO: Gene expression omnibus
