# Supplementary material for: CREB3L1 promotes tumor growth and metastasis of anaplastic thyroid carcinoma by remodeling the tumor microenvironment
Source: Mol Cancer. 2022 Oct 3;21:190. doi: 10.1186/s12943-022-01658-x (PMC9531463; doi:10.1186/s12943-022-01658-x)
Supplement: Supplementary file 1 — Additional file 1: Figure S1. CREB3L1 transcriptionally regulated the expression of COL5A1 to facilitate the aggressiveness of ATC. (A) The relationship of COL5A1 expression and tumor stages in thyroidcancer was analyzed. (B) Survival analysis of COL5A1 in thyroid cancer was analyzed. (C) IHC staining was used to analyze COL5A1 expression in NT, PTC and ATC tissues. (D) Transwell invasion assay was used to analyze the metastasis ability after the silence of COL5A1 in 8505C. (E-F) The AnimalTFDB3 database was used to predict the binding sequence of CREB3L1 to the COL5A1 promoter, and the Dual-luciferase assay to detect the transcriptional activation of CREB3L1 to COL5A1 wild-type or mutant binding sequence. (G) PredictProtein database was used to analyze the nuclear localization sequence of CREB3L1. Data shown are results of three independent experiments and shown as mean ± standard deviation(SD). *P < 0.05, **P < 0.01. [file 12943_2022_1658_MOESM1_ESM.docx]

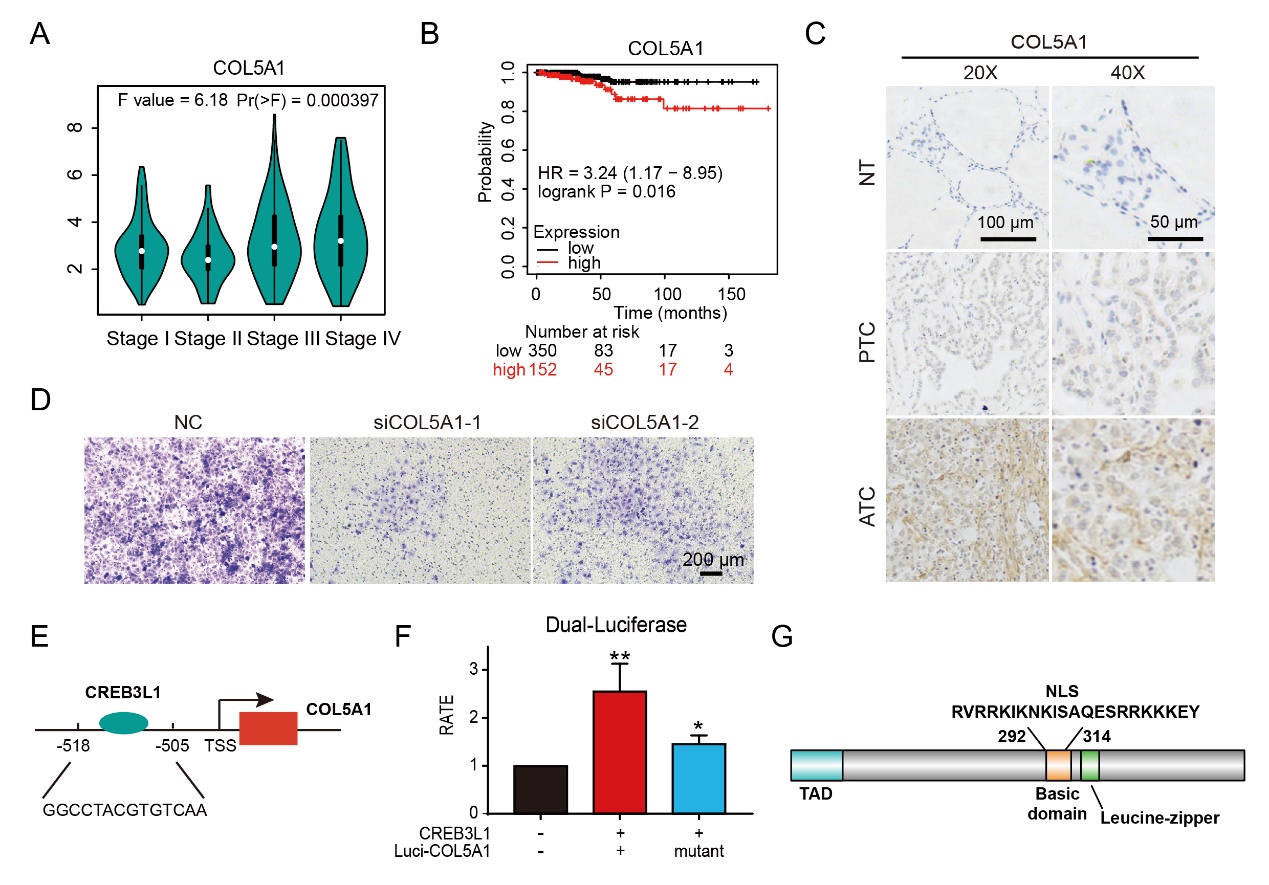


**Figure S1. CREB3L1 transcriptionally regulated the expression of COL5A1 to facilitate the aggressiveness of ATC.** (A) The relationship of COL5A1 expression and tumor stages in thyroid cancer was analyzed. (B) Survival analysis of COL5A1 in thyroid cancer was analyzed. (C) IHC staining was used to analyze COL5A1expression in NT, PTC and ATC tissues. (D) Transwell invasion assay was used to analyze the metastasis ability after the silence of COL5A1 in 8505C. (E-F) The AnimalTFDB3 database was used to predict the binding sequence of CREB3L1 to the COL5A1 promoter, and the Dual-luciferase assay to detect the transcriptional activation of CREB3L1 to COL5A1 wild-type or binding sequence mutant. (G) PredictProtein database was used to analyze the nuclear localization sequence of CREB3L1. Data shown are results of three independent experiments and shown as mean ± standard deviation (SD). **P* < 0.05, ***P* < 0.01.
