# Supplementary material for: CREB3L1 promotes tumor growth and metastasis of anaplastic thyroid carcinoma by remodeling the tumor microenvironment
Source: Mol Cancer. 2022 Oct 3;21:190. doi: 10.1186/s12943-022-01658-x (PMC9531463; doi:10.1186/s12943-022-01658-x)
Supplement: Supplementary file 2 — Additional file 2: Figure S2. Effect of CREB3L1 knockdown on the differentiation of CAFs. (A-B) Flow cytometry analysis of FAP or PDGFRα positive fibroblasts after co-culture with 8505C cells, respectively. CREB3L1 was knocked down in 8505C cells to evaluate its effect on the differentiation of CAFs. [file 12943_2022_1658_MOESM2_ESM.docx]

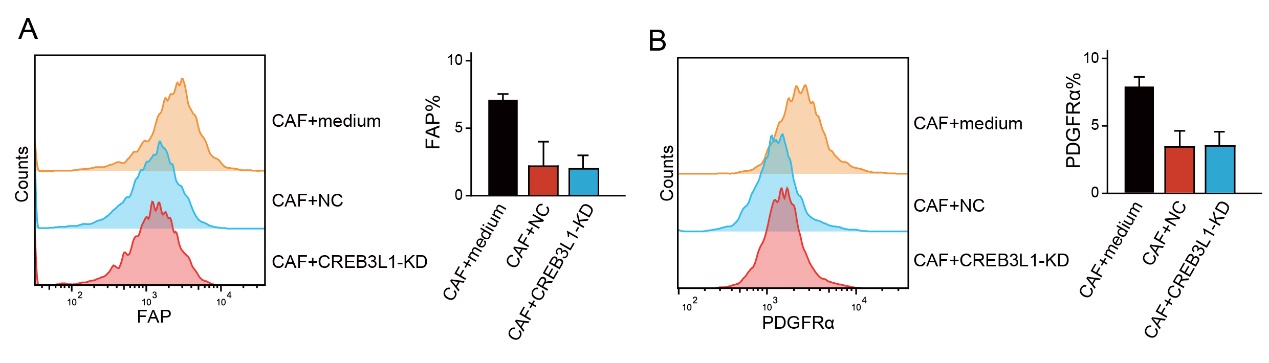


**Figure S2. Effect of CREB3L1 knockdown on the differentiation of CAFs.** (A-B) Flow cytometry analysis of FAP or PDGFRα positive fibroblasts after co-culture with 8505C cells, respectively. CREB3L1 was knocked down in 8505C cells to evaluate its effect on the differentiation of CAFs.
